# The Impact of Taking Care of Grandchildren on Health Outcomes in Japanese Community-Dwelling Elderly

**DOI:** 10.1093/geroni/igaa057.1123

**Published:** 2020-12-16

**Authors:** Yoshinori Fujiwara, Kumiko Nonaka, Masataka Kuraoka, Sachiko Murayama, Yuta Nemoto, Hiroshi Murayama, Yoh Murayama, Erika Kobayashi

**Affiliations:** 1 Tokyo Metropolitan Institute of Gerontology, Tokyo, Japan; 2 Tokyo Metropolitan Institute of Gerontology, Tokyo, Japan, Tokyo, Japan; 3 Tokyo Metropolitan Institute of Gerontology, Tokyo, Tokyo, Japan

## Abstract

Taking care of grandchildren may provide health benefits to older adults due to keeping their social roles and feeling more generative; however, we have scarce knowledge of the relationships in Asian countries. This study addressed this question in older Japanese. The data was obtained from a two-year follow-up mail survey conducted in 2016 on 3,116 randomly selected older Japanese, aged 65-84 years, living in a metropolitan area. The main outcome was deterioration of health assessed by the Self-Rated Health (SRH), WHO-5, and Instrumental Activities of Daily Living (IADLs), defined as decline in 1 or more points obtained after 2 years of follow-up. The frequency of taking care of grandchildren was assessed as every day, 4-6 days per week, 1-3 days per week, 1-3 days per month, several days per year, and none. A multiple linear regression examined the impact of taking care of grandchildren as a predictor of protection of decline in SRH, WHO-5 and IADLs. The models were adjusted for confounding factors. Of 1,561 who responded to the follow-up survey, 959 people had grandchildren at baseline. The subjects had a mean age of 73.2±5.3 years, and mean scores of SRH:2.1±0.6; WHO-5;16.1±5.3, IADLs; 4.9±0.6 (higher scores represent higher evaluation). The higher frequency of taking care of grandchildren were longitudinally associated with less decline in SRH, WHO-5, and IADLs (standardized partial regression coefficient, β=-0.090, p=0.013; β =-0.023, p=0.547; β =-0.107, p=0.008, respectively). In conclusion, taking care of grandchildren might be a protective factor of comprehensive and functional health deterioration.

